# Prevalence and contributing factors of executive cognitive dysfunction symptoms in university students

**DOI:** 10.1371/journal.pone.0323783

**Published:** 2025-06-09

**Authors:** Mohammad Fraiwan, Fidaa Almomani, Hanan Hammouri

**Affiliations:** 1 Department of Computer Engineering, Faculty of Computer and Information Technology, Jordan University of Science and Technology, Irbid, Jordan; 2 Department of Rehabilitation Sciences, Faculty of Applied Medical Sciences, Jordan University of Science and Technology, Irbid, Jordan; 3 Department of Mathematics and Statistics, Faculty of Science and Arts, Jordan University of Science and Technology, Irbid, Jordan; National Institutes of Health, University of the Philippines Manila / De La Salle University, PHILIPPINES

## Abstract

The importance of executive cognition should not be overlooked in the private and academic lives of university students. It includes important constituents of the human mind, including but not limited to, organizing, directing, solving problems, and controlling oneself and these processes are central to surviving the rigors of higher education. Good executive function enables the students to perform complex tasks, such as fighting deadlines, understanding the course structure, and participating in many other activities. Further, it assists in arriving at resolutions and managing tensions as one transitions into adulthood, both of which are critical. In other words, executive cognitive deficits are correlated with problems in academic progression, time management, and overall adjustment to the possible social and emotional stressors of university experience. This cross-sectional study, involving 1,204 students, used the validated Arabic version of the Dysexecutive Questionnaire (DEX) to measure executive cognitive function, along with demographic and lifestyle data. The results showed significant associations between executive cognition dysfunction and certain lifestyle factors common among generation Z, such as hours spent on smartphones or electronic devices (p < 0.0001), social media platform use (p = 0.0484), weekly fast food consumption (p < 0.0001), and daily hours on social media (p < 0.0001). Additional factors included weak family relationships (p = 0.0018), gender (p = 0.029), family income (p = 0.0164), urban residence (p = 0.0176), prior mental health consultations (p < 0.0001), and parental separation (p < 0.0375). Conversely, regular sports participation and exercise were linked to lower dysfunction scores (p = 0.0327), suggesting a protective effect. These findings underscore the impact of lifestyle and personal circumstances on cognitive functioning, highlighting the need for balanced technology use, healthy diets, strong family and social networks, and physical activity. Early psychological support for at-risk students may further enhance cognitive resilience and overall well-being.

## Introduction

Young adulthood (18–25 years) is a critical developmental stage marked by significant transitions and responsibilities, particularly for university students who must navigate academic, social, and personal challenges. Success in higher education requires strong executive cognition, which encompasses skills such as planning, problem-solving, task management, and self-regulation [1]. These abilities enable students to prioritize tasks, meet deadlines, and balance commitments while fostering adaptability and resilience in demanding environments [2,3]. However, deficits in executive cognition (e.g., difficulties in organization, attention, and impulse control) can contribute to academic underperformance, procrastination, and heightened stress [4]. Beyond academics, executive functioning influences broader aspects of development, including career readiness, personal growth, and relationship management [5]. Supporting these cognitive skills through educational interventions, mental health resources, and healthy lifestyle habits can enhance students’ ability to manage university demands and promote long-term success [6].

Executive dysfunction in university students can manifest in various ways, including poor time management, chronic procrastination, and difficulty adapting to new information [4]. Cognitive inflexibility may hinder problem-solving, while impulsivity can lead to distractions and poor decision-making. Emotional dysregulation, characterized by stress intolerance and frustration, may negatively impact mental health and social relationships [7]. Additionally, memory and organizational difficulties can impair academic performance and overall life satisfaction. Recognizing these challenges is of paramount importance for developing effective interventions that support students in overcoming cognitive difficulties.

Addressing executive dysfunction requires targeted interventions, including cognitive-behavioral therapy (CBT) to enhance time management and problem-solving skills. Universities can implement mental health initiatives, screening programs, and access to cognitive training services. Lifestyle adjustments, such as promoting physical activity, adequate sleep, and stress management strategies, have also been shown to improve executive function. Academic accommodations like extended deadlines, alternative exam formats, and structured mentoring programs can further support affected students. By implementing these strategies, universities can foster an inclusive academic environment that enables all students to reach their full potential. The present study aims to examine the prevalence and contributing factors of executive dysfunction symptoms among university students. This work will eventually help guide these targeted interventions.

Several tools exist for assessing neurocognitive impairments, with the Dysexecutive Questionnaire (DEX) being particularly effective in evaluating inhibition, intention, social regulation, and abstract problem-solving [8]. Initially designed as a qualitative measure of daily life symptoms, the DEX has also demonstrated utility in nonclinical populations for assessing executive function. Research suggests that rather than serving as a singular measure of executive dysfunction, the DEX is best understood through its subscales, each targeting distinct cognitive constructs [9]. This multidimensional perspective aligns with prior factor analyses indicating variations in subscale distinctions across studies [8,10–12]. Furthermore, this approach supports theoretical models proposing that executive function comprises multiple interrelated sub-processes [13].

The remainder of the paper is organized as follows. The Methods section provides an overview of the ethical approvals and declarations, followed by a description of the study design, participant selection, and sampling strategy. It also outlines the procedures followed, the data collection instruments used, and the translation and reliability processes employed to ensure consistency across measures. Furthermore, this section discusses the data processing methods and the statistical analyses applied. The Results and Discussion section presents the key findings and relates them to existing literature, emphasizing their implications and suggesting potential directions for future research.

## Materials and methods

### Ethical approval declarations

The Institutional Review Board (IRB) at King Abdullah University Hospital and the Deanship of Scientific Research at Jordan University of Science and Technology in Jordan granted approval for the present study (Ref. 99/11/2018). All procedures adhered to the principles outlined in the Declaration of Helsinki, and written informed consent was secured from each participant prior to their involvement in this study. The study did not involve minors.

### Design

This study utilized a cross-sectional design and employed a descriptive-correlational approach to examine variables associated with symptoms of executive cognitive dysfunction among university students. The recruitment started on October 1st, 2022 and ended on December 31st, 2023.

### Participants

Participants for this study were drawn from three public universities–Yarmouk University, Jordan University of Science and Technology (JUST), and Tafila Technical University–and two private institutions, Irbid University and Jadara University. While all of the universities, except Tafila Technical University, are located in northern Jordan, Tafila is situated in the southern part of the country. Together, these five universities have a total student population of approximately 80,000.

Students were included in this study if they were enrolled full time in any of the five universities, completed at least one semester of university study, and were actively registered for classes during the data collection semesters. Students were excluded if they had a history of severe psychiatric disorders, chronic diseases (e.g., cancers, severe autoimmune diseases, severe neurological disorders), or physical disabilities.

### Sample

The study utilized a representative sample of students from all academic levels–freshmen, juniors, seniors, and final-year students–across the five universities. To determine the necessary sample size, a power analysis was conducted using the Cochran’s method for finite populations, see Eq 1. Thus, the necessary sample size (*n*_*finite*_) for an estimated student population of 80,000 with a 95% confidence level and a 5% margin of error is approximately 383 respondents.

$$n_{finite}=\frac{nN}{N+n-1}$$ (1)

Where:

$n=\frac{Z^{2}p(1-p)}{e^{2}}$

N = 80,000 (population size)

Z = 1.96 (Z-score for 95% confidence level)

p = 0.5 (proportion, set to 0.5 for maximum variability)

e = 0.05 (margin of error, 5%)

### Procedure

After securing approval from the IRB committee and the Deanship of Research at JUST, the researchers randomly selected courses with large class sizes (over 80 students per section) to facilitate the efficient collection of a substantial number of responses and to enhance response rates. With the written consent of the instructors, the researchers introduced the study, outlining its objectives and the content of the survey. The questionnaires were distributed electronically using a QR code that directed students to a Google Forms survey. Prior to submitting the questionnaire, students were further required to provide electronic consent by signing an informed consent form. Around 5,000 students across the five universities were invited to participate in the electronic survey.

Participants were requested to complete a survey that explored various socio-demographic factors, including age, gender, nationality, and place of residence (rural or urban), as well as aspects of family and social support, the number of siblings, and monthly family income. The survey also examined potential risk factors associated with dysexecutive functioning in relation to executive cognition. These risk factors included body mass index (BMI), determined from participants’ weight and height, the type of university attended (private or public), academic level and major, grade point average (GPA), the frequency of part-time employment while studying, weekly exercise hours, daily smartphone usage, weekly engagement with electronic games, the number of social media accounts, daily social media use, weekly fast food consumption, and any prior consultations with psychiatrists or psychologists. The selection of these factors was informed by an analysis of the student body across the five universities, leveraging the authors’ combined academic experience of over 50 years in this educational landscape. In addition, these variables were drawn from the authors’ own published works [14] and the broader literature on dysexecutive function and related constructs [15]. This approach ensured that the selected variables were both contextually relevant and theoretically grounded.

### Instrument

In this study, the validated Arabic version of the DEX revised (DEX-R) executive cognition subscale was employed. The short Arabic version of the DEX-R demonstrated a Cronbach’s alpha of 0.943, indicating excellent internal consistency, along with strong test-retest reliability (ICC=0.97). The executive cognition subscale consists of a checklist of symptoms rated on a 5-point Likert scale, ranging from (1) “never" to (5)“very often." This subscale possesses robust psychometric properties and is available in two formats: a self-report version, which was used in this research, and an independent-rating version. Initially, the dysexecutive questionnaire was developed as a supplementary tool for the behavioral assessment of dysexecutive syndrome (BADS) [16], aiming to identify observable changes in executive dysfunction following acquired brain injuries. It encompasses items that evaluate various facets of executive functioning, such as abstraction, impulsivity, confabulation, planning skills, mood regulation, and decision-making. The questionnaire addresses a wide array of specific concerns, including difficulties with attention, memory issues, information processing, behavioral control, emotion regulation, and self-awareness [8,17]. However, DEX utility has since been extended to non-clinical populations, including healthy individuals and those with subclinical executive functioning challenges [18]. Chan *et al*. [19] showed that the DEX could serve as a tool for assessing executive functions in individuals not suffering from acquired brain injuries, psychiatric conditions, or personality disorders.

Originally designed as a qualitative assessment tool for customizing rehabilitation to individual challenges, the DEX has evolved into a quantitative tool for diagnostic purposes [20,21]. According to Simblett and Bateman [9], the DEX captures multiple latent variables that represent different dimensions of executive dysfunction. The focus of this study is on applying the executive cognition subscale of the DEX-R within the Jordanian university population. A summary of the elements comprising this executive cognition subscale from the DEX-R is presented in Table 1. The executive cognition subscale was translated into Arabic, incorporating cultural adaptations to align with the nuances of the Arabic language.

**Table 1:** Summary of the executive cognition subscale elements from the DEX-R based on Stuss [13].

Item No. (DEX-R)	Item Name in (DEX-R)
2	Prospective memory problems
5	Planning problems
9	Poor verbal fluency
13	Abstract thinking problems
17	Working memory problems
21	Temporal sequencing problems
25	Poor organisational ability
29	Distractibility
34	Information processing problems
36	Complex attention problems

#### Translation and reliability procedure.

The executive cognition subscale was translated into Arabic in accordance with established guidelines [8,17,20,22] and the framework prescribed by Sperber *et al*. [23] for translation and adaptation of instruments. Three bilingual occupational therapists independently translated the items of the DEX-R into Arabic. Any discrepancies in the translations were resolved through discussion, ultimately arriving at a consensus on a final version. This version was then refined based on feedback gathered from a pilot test involving 65 participants. A back-translation was conducted by a bilingual translator who is a native English speaker from the United States, fluent in Arabic, and residing in Jordan. A group of 10 researchers evaluated and compared the original items with the back-translated items to assess their similarity. A cut-off score of 0.8 or higher (on a scale where 0 indicates “not similar” and 1 indicates “similar”) was employed to evaluate the adequacy of the Arabic translation. This threshold indicated that at least 80% of the evaluators agreed that the back-translated item closely resembled the original. Scores below 0.8 suggested potential issues in the interpretation of the translation. In total, 90 participants aged 18 to 25 (mean age = 21.7 years ± 2.9) were involved in this cross-sectional study, which aimed to assess the reliability of the subscale. The sample was randomly selected over a 6-month period , encompassing a diverse range of socioeconomic statuses to represent the general population’s distribution.

To ensure cultural and linguistic accuracy in the Arabic adaptation of the instrument, slight modifications were made to specific items for clarity and consistency with their English counterparts. To this end, the literal translation for the DEX questions 5, 15, and 18 was slightly adjusted, as follow: Question 5 includes the expression ‘over the top’ to indicate exaggeration; in Arabic, a literal translation would suggest a spatial relationship rather than an intensifier, so it was adapted to convey the intended meaning. Similarly, question 15 uses the term ‘restless’, which, in this context, refers to continuous movement (i.e., physical restlessness) rather than a lack of rest. Additionally, question 18 includes the phrase ‘keep his/her mind on something’, meaning concentration, which required adaptation to ensure the intended interpretation was preserved. In general, the instrument questions have simple straightforward wording and no further modifications were made.

### Data processing and statistical analyses

**Fig 1:**
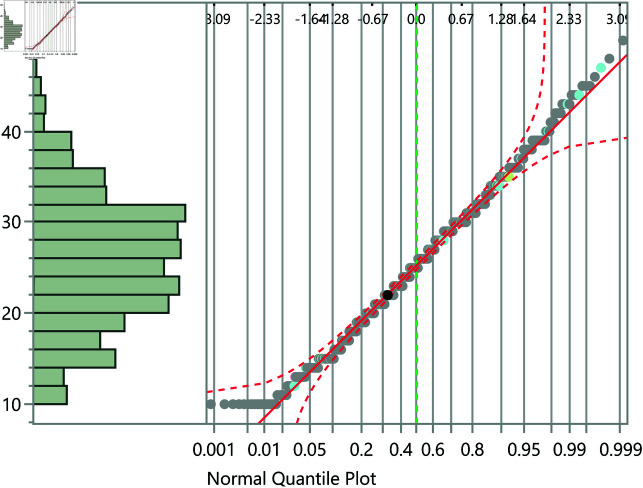
The Q-Q plot confirming the normal distribution of the executive cognition subscale score.

The data were analyzed using JMP statistical software [24]. Initially, statistical summaries were generated for the dependent variable and significant factors. For independent categorical variables, the t-test was utilized to compare the means between two groups, while one-way analysis of variance (ANOVA) was conducted for variables with more than two groups. To further interpret statistically significant differences (i.e., *p*<0.05), Tukey’s honestly significant difference (HSD) test was applied. Since this test assumes homogeneity of variances across groups, Levene’s test was used to confirm this assumption. For continuous independent variables, linear regression analysis was employed. These statistical methods necessitate that the executive cognition score (the outcome variable) be normally distributed, which was confirmed using quantile-quantile (Q-Q) plots, as illustrated in Fig 1.

**Fig 2:**
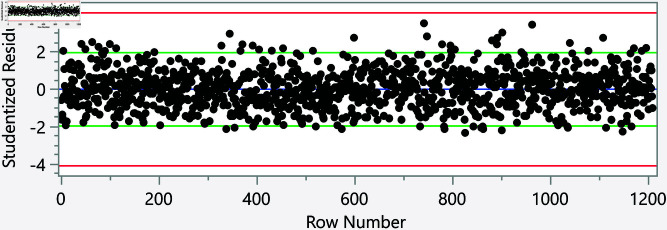
Externally studentized residuals with 95% simultaneous limits (Bonferroni) in red, individual limits in green for the number of daily hours spent on social media.

**Fig 3:**
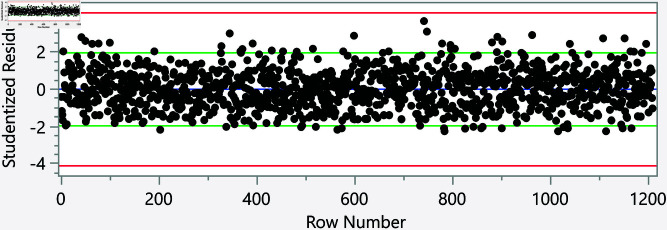
Externally studentized residuals with 95% simultaneous limits (Bonferroni) in red, individual limits in green for the number of weekly times eating fast food.

**Fig 4:**
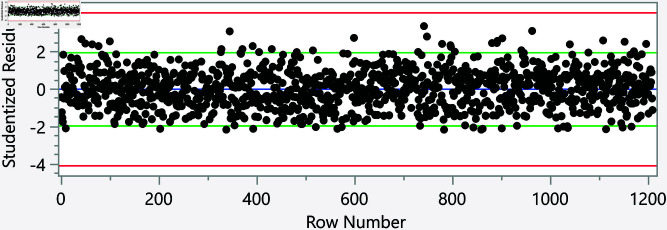
Externally studentized residuals with 95% simultaneous limits (Bonferroni) in red, individual limits in green for the number of daily hours using smartphone/electronic devices.

**Fig 5:**
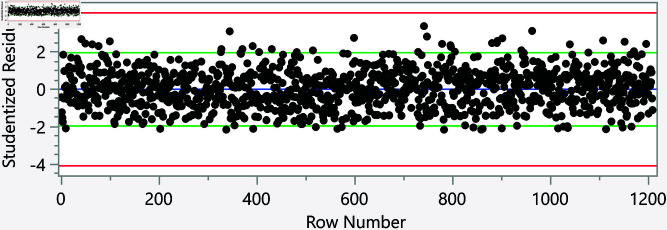
Externally studentized residuals with 95% simultaneous limits (Bonferroni) in red, individual limits in green for the number of weekly hours exercising/playing sports.

**Fig 6:**
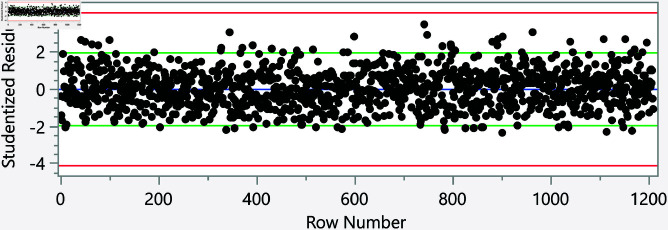
Externally studentized residuals with 95% simultaneous limits (Bonferroni) in red, individual limits in green for the number of social media platforms used.

In the assessment of homoscedasticity within our regression analysis, we examined the distribution of externally studentized residuals across predicted values of executive cognition. The regression plots incorporated 95% simultaneous limits, using the Bonferroni method, and individual limits to provide an evaluation of variance consistency, see Figs 2–6. The residuals, highlighted in red for the simultaneous limits and green for the individual limits, did not exhibit patterns deviating significantly from these thresholds. This consistency across the range of predicted values indicates that the assumption of homoscedasticity is upheld in our data. Such findings confirm that the variance of errors in our regression model is constant, which indicates that the model’s estimates are both efficient and unbiased. The absence of heteroscedasticity enhances the reliability of the regression analysis, reinforcing the validity of the conclusions drawn about the factors influencing executive cognition.

## Results

Counts and percentages were calculated for the categorical variables, as shown in Table 2. The demographic analysis indicates that a significant majority of respondents are Jordanian (92%), with 61% residing in urban areas. The gender distribution is nearly equal, consisting of 61% female and 39% male participants. Most individuals come from intact families (97%) and maintain strong connections with extended family members (67%). The sample predominantly consists of students from public universities (92%), with a considerable proportion (61%) belonging to families earning less than 1000 JOD per month. Nearly all participants are undergraduates (99%), with the largest segment being in their second year (51%). The academic performance levels are varied, with 41% achieving a GPA in the B/B- range. Social media usage is almost universal, reported by 99% of participants, and a large majority (86%) live with their parents. A small percentage (7%) have sought help from a psychologist or psychiatrist, while many (64%) do not engage in work while studying.

**Table 2:** Summary of categorical socio-demographic and risk factors statistics.

Variable	Value	Count	Percentage
Nationality	Other	93	8%
	Jordan	1111	92%
Area of living	Rural	467	39%
	City	737	61%
Sex	Male	466	39%
	Female	738	61%
Parents Divorced?	No	1168	97%
	Yes	36	3%
Strong relationship with extended family?	No	398	33%
	Yes	806	67%
University	Private	95	8%
	Public	1109	92%
Family monthly income (JOD^1^])	<1000	731	61%
	1000-2000	333	28%
	2001-3000	70	6%
	>3000	70	6%
Undergraduate	Yes	1186	99%
	No	18	1%
Academic level (year)	1	97	8%
	2	612	51%
	3	294	24%
	4	201	17%
GPA	< C-	78	6%
	C,C-	270	22%
	B,B-	499	41%
	A,A-	357	30%
Work and Study?	Never	776	64%
	Sometimes	224	19%
	Most of the times	95	8%
	Always	109	9%
Do you have a social media account?	No	14	1%
	Yes	1190	99%
Do you live with parent(s)?	No	167	14%
	Yes	1037	86%
Previous visits to a psychologist/psychiatrist?	No	1116	93%
	Yes	88	7%

^1^ JOD = 1.41 USD (fixed rate).

The means and standard deviations were computed for the continuous variables (Table 3). These variables highlight aspects of lifestyle, physical health, and cognition. On average, participants have about 4.5 siblings ± 1.924, indicating larger family sizes. Their average weight is 65.97 kg ± 15.03 and height is 167.48 cm ±9.81, with an average BMI of 23.39 ± 4.22, which falls within the healthy range. Participants spend a significant amount of time on social media daily (5.12 hours ±4.11 and use smartphones or electronic devices for an average of 7.08 hours per day ± 4.30. Weekly, they exercise for about 3.8 hours ±5.51 but also consume fast food 2.1 times ±1.44. Interestingly, they spend less time on electronic games, with a weekly average of 2.22 hours ±5.27. Finally, the executive cognition subscale score averages 25.32 ±7.21.

**Table 3:** Summary of continuous socio-demographic and risk factors statistics, along with total DEX-R score for executive cognition elements.

Variable	Mean	±SD
Number of siblings	4.547	1.924
Weight (kg)	65.972	15.025
Height (cm)	167.480	9.806
BMI (calculated)	23.393	4.224
Daily hours spent on social media	5.122	4.105
Weekly hours spent exercising	3.802	5.512
Weekly times eating fast food.	2.104	1.444
Daily hours spent on smartphone/electronic devices	7.082	4.296
Weekly hours spent on electronic games	2.217	5.273
DEX-R executive cognition score	25.316	7.211

Varied levels of executive cognitive functioning among participants were observed, see Tables 4 and 5 . The prevalence of normal cognitive traits was found to be 21.51%, which was the lowest percentage. Mild dysfunction symptoms were more prevalent at 24.17%. Furthermore, moderate symptoms were observed in 25.42% of participants, which reflects a potential group being at-risk of deteriorating and developing further strong symptoms. Notably, a substantial proportion, 28.90%, demonstrated strong cognitive dysfunction symptoms, which is the highest group percentage of all. In general, more than half of the subjects require some level of professional help. This detailed prevalence landscape indicates a serious existing execution cognition problem that is at great danger of expanding if no appropriate interventional measures are taken by all stakeholders.

**Table 4:** The prevalence of the four levels of executive dysfunction symptoms.

Score	Count	Prob
< 20 (normal)	259	0.21512
20-24 (mild)	291	0.24169
25-29(moderate)	306	0.25415
≥30 (strong)	348	0.28904

**Table 5:** The calculated clinical quantiles for the executive cognition subscale score. The minimum score was 10 and maximum was 50.

Quartile	Subscale score
2.5%	12
10%	15
25% (mild)	20
50% (moderate)	25
75% (strong)	30
90%	35
97.5%	39.875
99.5%	45

**Fig 7:**
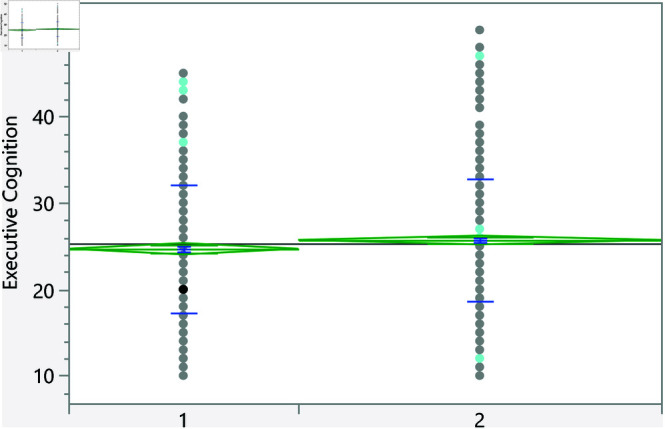
Dot plot of the executive cognition score versus area of living (1 represent rural). The score was significantly higher for students living in urban areas.

**Fig 8:**
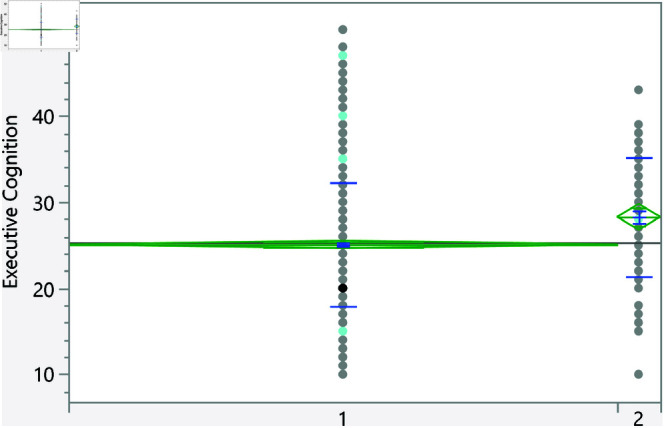
Dot plot of the executive cognition score versus having visited a psychiatrist/psychologist (1 represent no). Those with prior visits had a higher score on average.

**Fig 9:**
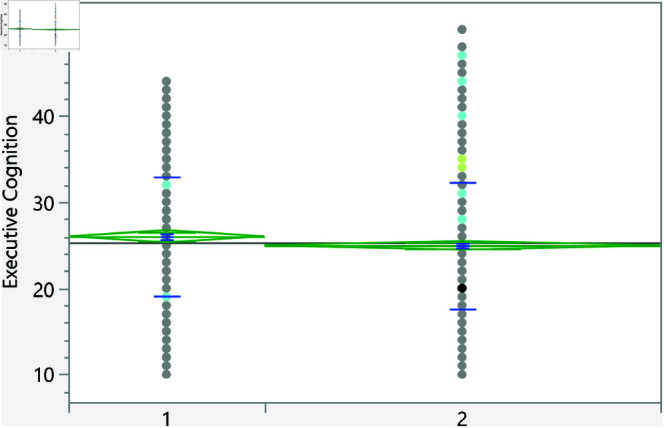
Dot plot of the executive cognition score versus having strong relationship with extended family (1 represents no, and higher mean score).

**Fig 10:**
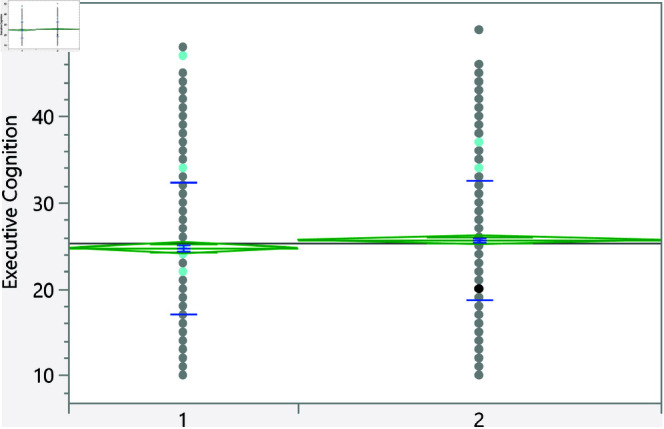
Dot plot of the executive cognition score versus the gender (1 represent male, and lower mean score).

**Fig 11:**
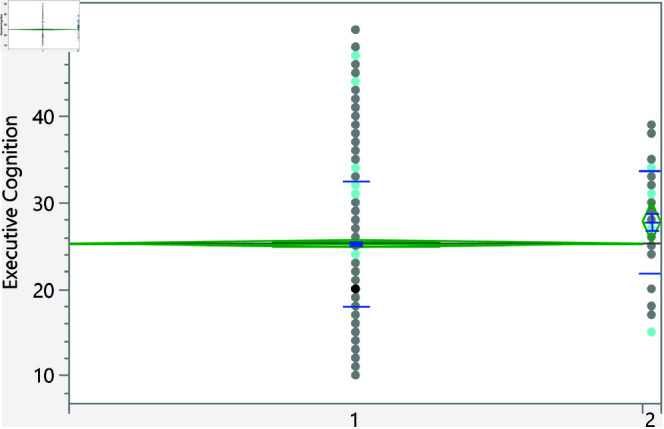
Dot plot of the executive cognition score versus parents being divorced (1 represent no, and lower mean score).

**Fig 12:**
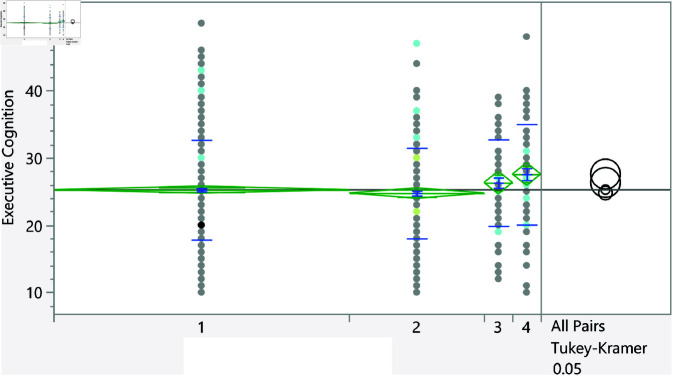
Dot plot of the executive cognition score versus the family income. Significant differences between level 2 and 4. Level with higher income had higher score on average.

**Fig 13:**
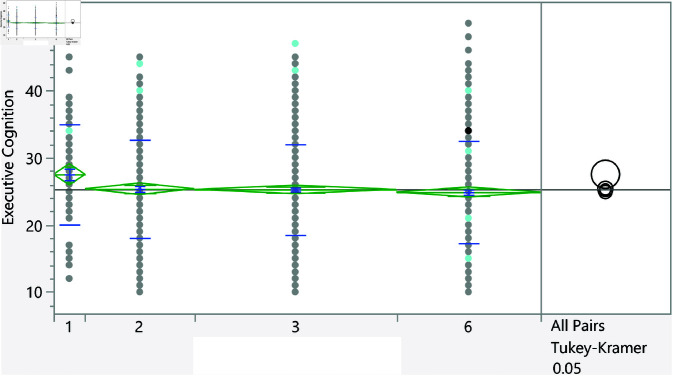
Dot plot of the executive cognition score versus the GPA (1 represent < C-), 6 represent (A,A-). Lowest GPA had significantly higher score in comparison to the highest GPA.

Based on the t-test, the categorical variables listed in Table 6 significantly affect the executive cognition score. Furthermore, Figs 7–13 show the distribution of the executive cognition score versus these factors. The Levene’s test indicated that the assumption of homogeneity of variances was not violated for any variable, with p = 0.0879 for GPA groups and p = 0.0922 for family income groups. In addition, to address the problem of multiple comparisons, the Holm-Bonferroni method was used to adjust the significance levels to control the family-wise error rate (FWER). The adjusted significance threshold for each variable is shown in Table 6. The p-value for previous visits to a psychologist or psychiatrist is <0.0001, and individuals without such visits have a mean score of 25.08 ± 7.18, while those who have sought psychological support score higher at 28.27 ± 6.90. The area of living also shows significance with a p-value of 0.0176; rural residents scored 24.70 ± 7.40 compared to urban residents at 25.71 ± 7.06. Additionally, a strong relationship with extended family yields a p-value of 0.018, with lower scores for those reporting such relationships at 25.00 ± 7.33, while individuals without strong family ties scored 26.02 ± 6.91. Gender differences were noted with a p-value of 0.029, showing females at 25.68 ± 6.92 outperforming males at 24.74 ± 7.62. The variable of parental marital status indicates that individuals from divorced families scored higher at 27.78 ± 5.93 compared to those from intact families (25.24 ± 7.23), and family income shows significant variation (p = 0.0164), with higher incomes correlating with better executive functioning. Finally, GPA analysis revealed a significant relationship (p = 0.0351), with lower scores among those achieving lower GPAs (e.g., 27.5 ± 7.47 for GPA < C-) compared to those with higher GPAs, underscoring the influence of academic performance on executive cognition.

**Table 6:** Summary of significant dichotomous and ordinal categorical variables along with their effect sizes. The effect sizes for GPA and family income were measured using $\eta^{2}$, while Cohen’s d was used for the remaining variables.

Variable	P-value	adj α	Value	Mean	± SD	Statistic	Value
Previous visits to a psychologist/psychiatrist?	<0.0001	0.01	No	25.08	7.18	t(1202)	4.0210
			Yes	28.27	6.90		
Area of living^1^	0.0176	0.0125	Rural	24.70	7.40	t(1202)	2.3780
			Urban	25.71	7.06		
Strong relationship with extended family?^1^	0.018	0.0167	No	26.02	6.91	t(1202)	-2.3695
			Yes	25.0	7.33		
Gender^1^	0.029	0.025	Male	24.74	7.62	t(1202)	2.1865
			Female	25.68	6.92		
Divorced parents?	0.0375	0.05	No	25.24	7.23	t(1202)	2.0830
			Yes	27.78	5.93		
Family income	0.0164 (levels 4 vs 2)	0.025	<1 K	25.26	7.42	F(3, 1200)	3.4031
			1K-2K	24.76	6.74		
			2K-3K	26.3	6.45			
			>3K	27.56	7.48			
GPA	0.0351 (highest vs lowest)	0.05	<*C*–	27.5	7.47	F(3, 1200)	2.8750
			C,C-	25.36	7.32		
			B,B-	25.3	6.76		
			A,A-	24.88	7.62		

^1^Lost significance post Holm-Bonferroni adjustment.

**Fig 14:**
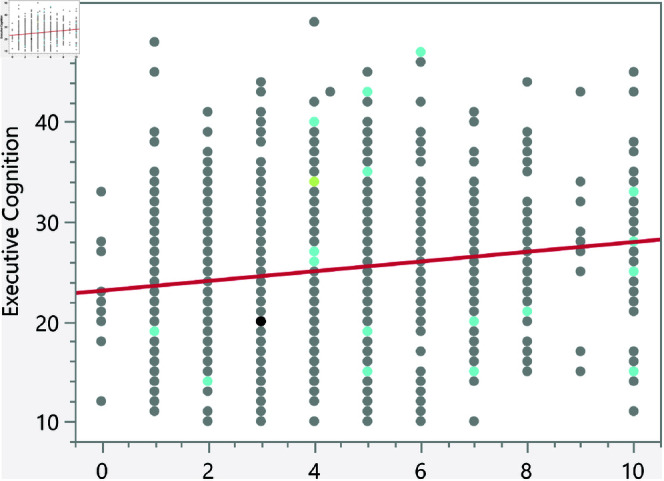
Bivariate fit of the executive cognition score versus number of daily hours spent on social media.

**Fig 15:**
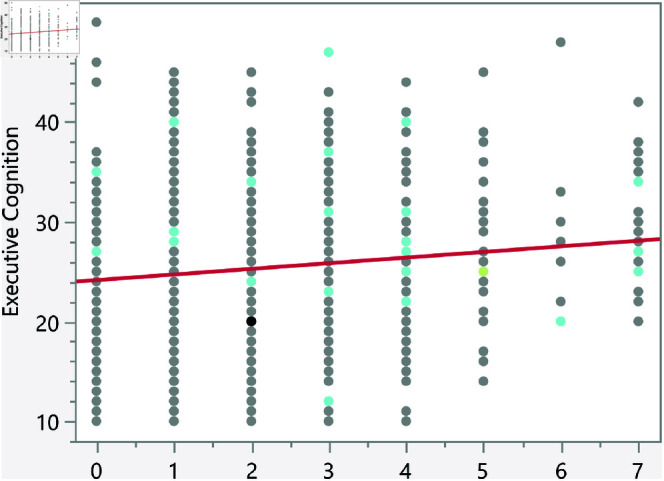
Bivariate fit of the executive cognition score versus number of weekly times eating fast food.

**Fig 16:**
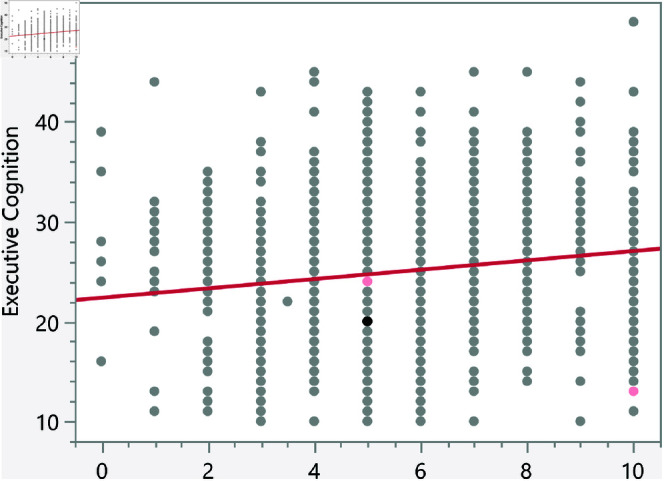
Bivariate fit of the executive cognition score versus number of daily hours using smartphone/electronic devices.

**Fig 17:**
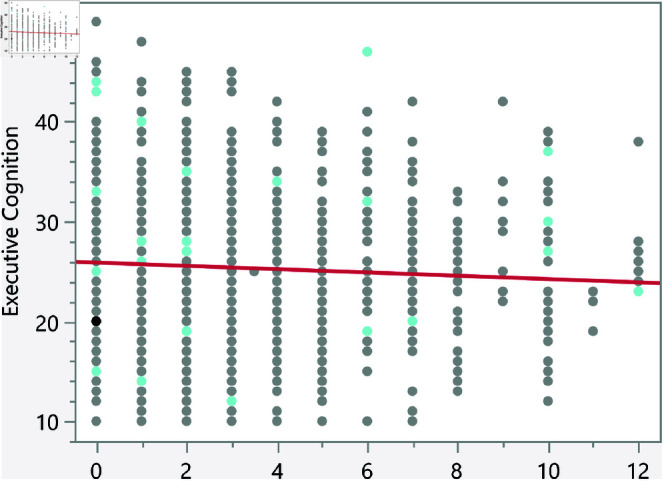
Bivariate fit of the executive cognition score versus number of weekly hours exercising/playing sports.

**Fig 18:**
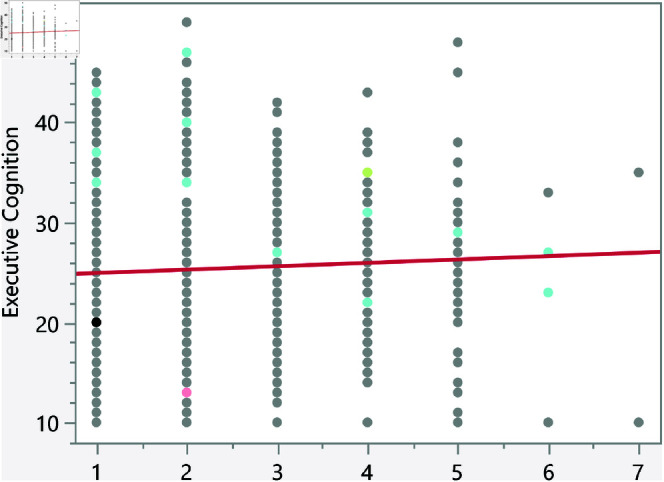
Bivariate fit of the executive cognition score versus number of social media platforms used.

The regression results indicate several significant relationships between lifestyle factors and executive cognition scores. Table 7 presents the significant continuous factors along with their p-values and prediction formulas. In addition, Figs 14–18 show the bivariate fit of the executive cognition score versus these factors. All factors, except for the number of weekly hours spent exercising or playing sports, show a positive relationship with the executive cognition score, suggesting that as these factors increase, the score also increases. The number of social media platforms used (p = 0.0484) and daily hours spent on social media (p < 0.0001) both show positive associations with executive cognition, with increases of 0.34 and 0.49 points, respectively, for each additional platform or hour spent. In contrast, weekly hours exercising/playing sports (p = 0.0327) show a negative association, reducing the score by 0.16 points per hour. Additionally, the number of weekly fast-food meals (p < 0.0001) and daily hours using electronic devices (p < 0.0001) positively impact executive cognition, increasing the score by 0.56 and 0.47 points, respectively. These results suggest that certain lifestyle factors contribute both positively and negatively to executive cognitive function.

**Table 7:** Summary of significant continuous variables. *x* is the corresponding variable in each line.

Variable	P-value	Prediction equation
Number of social media platforms used	0.0484	24.58+0.34×x
Daily hours spent on social media	< 0.0001	23.06+0.49×x
Number of weekly hours exercising/playing sports^1^	0.0327	25.88−0.16×x
Number of weekly times eating fast food	< 0.0001	24.13+0.56×x
Number of daily hours using smartphone/electronic devices	< 0.0001	22.37+0.47×x

^1^Lost significance post Holm-Bonferroni adjustment.

In a similar manner to the categorical variables, the Holm-Bonferroni method was used to control the FWER. The p-values were first ordered from the smallest to the largest as follows: Daily hours spent on social media (<0.0001), Number of weekly times eating fast food (<0.0001), Number of daily hours using smartphone/electronic devices (<0.0001), Number of weekly hours exercising/playing sports (0.0327), and Number of social media platforms used (0.0484). Each p-value was sequentially compared to progressively stringent alpha thresholds and adjusted according to its rank in this ordered list. Specifically, the smallest p-value was compared to α/5, the next smallest to α/4), and so forth, down to the largest p-value, which was compared against α/1. After implementing this correction, almost all of the variables continued to demonstrate significant p-values with the dependent variable. However, the variable Number of weekly hours exercising/playing sports, while initially significant, did not maintain this status post-adjustment. This stepwise approach of comparing each p-value to a more stringent criterion as we move through the ordered list ensures a stringent control of Type I errors.

Additional statistical analysis was conducted to assess the potential factors associated with executive cognition dysfunction. This involved calculating the 25th, 50th, and 75th percentile thresholds. The cutoff scores for the scales, derived from factor analysis, along with the statistical test results, are summarized in Tables 5 and 8. Quartiles are particularly valuable in understanding where an individual’s score ranks relative to others, especially in population-specific studies, where group variability plays a role. In epidemiological research, they are useful for analyzing risk distribution and offering context to identify outliers or extremes, thereby supporting more targeted interventions. Despite their utility, quartiles can lack precision and may differ depending on the population studied. While total scores are essential for assessing absolute severity in clinical diagnoses, quartiles are better suited for public health and population studies, helping to identify relative risk and highlight high-risk subgroups.

**Table 8:** Clinical quartile scores distribution between groups for the executive cognition subscale.

Variable	Value	Q1 (normal)	Q2 (mild)	Q3 (moderate)	Q4 (strong)	$\chi^{2}$	P-Value
Nationality	Other	18 (19.4%)	17 (18.3%)	31 (33.3%)	27 (29.0%)	5.739	0.125
	Jordan	241 (21.7%)	289 (26.0%)	260 (23.4%)	321 (28.9%)		
Area of living	Rural	128 (27.4%)	121 (25.9%)	91 (19.5%)	127 (27.2%)	20.1	0.0002
	City	131 (17.8%)	185 (25.1%)	200 (27.1%)	221 (30.0%)		
Sex	Male	122 (26.2%)	114 (24.5%)	108 (23.2%)	122 (26.2%)	10.235	0.0167
	Female	137 (18.6%)	192 (26.0%)	183 (24.8%)	226 (30.6%)		
Parents Divorced?	No	255 (21.8%)	294 (25.2%)	286 (24.5%)	333 (28.5%)	6.496	0.08
	Yes	4 (21.5%)	12 (33.3%)	5 (13.9%)	15 (28.9%)		
Strong relationship with	No	72 (18.1%)	105 (26.4%)	95 (23.9%)	126 (31.7%)	5.036	0.169
extended family?	Yes	187 (23.2%)	201 (24.9%)	196 (24.3%)	222 (27.5%)		
University	Private	20 (21.1%)	23 (24.21%)	17 (17.9%)	35 (36.8%)	3.998	0.2617
	Public	239 (21.6%)	283 (25.5%)	274 (24.7%)	313 (28.2%)		
Family income	< 1000	171 (23.4%)	182 (24.9%)	162 (22.2%)	216 (29.6%)	21.16	0.012
	1000-2000	72 (21.6%)	80 (24.0%)	99 (29.7%)	82 (24.6%)		
	2001-3000	9 (12.9%)	23 (32.9%)	15 (21.4%)	23 (32.9%)		
	> 3000	7 (10%)	21 (30%)	15 (21.4%)	27 (38.6%)		
Undergraduate	Yes	258 (21.8%)	301 (25.4%)	284 (24.0%)	343 (28.9%)	3.849	0.2783
	No	1 (5.6%)	5 (27.8%)	7 (38.9%)	5 (27.8%)		
Academic level	1	25 (25.8%)	24 (24.7%)	19 (19.6%)	29 (29.9%)	9.325	0.4078
(year)	2	138 (22.6%)	157 (25.7%)	144 (23.5%)	173 (28.3%)		
	3	53 (18.0%)	75 (25.5%)	69 (23.5%)	97 (33.0%)		
	4	43 (21.4%)	50 (24.9%)	59 (29.4%)	49 (24.4%)		
GPA	< C-	11 (14.1%)	22 (18.2%)	15 (19.2%)	30 (38.5%)	15.039	0.899
	C,C-	63 (23.3%)	73 (27.0%)	50 (18.5%)	84 (31.1%)		
	B,B-	101 (20.2%)	124 (24.9%)	139 (27.9%)	135 (27.1%)		
	A,A-	84 (23.5%)	87 (24.4%)	87 (24.4%)	99 (27.7%)		
Work and Study?	Never	162 (20.9%)	199 (25.6%)	197 (25.4%)	218 (28.1%)	13.175	0.1549
	Sometimes	51 (22.8%)	57 (25.5%)	58 (25.9%)	58 (25.9%)		
	Most times	18 (19.0%)	29 (30.5%)	18 (19.0%)	30 (31.6%)		
	Always	28 (25.7%)	21 (19.3%)	18 (16.5%)	42 (38.53%)		
Do you have a	No	1 (7.1%)	4 (28.6%)	4 (28.6%)	5 (35.7%)	1.756	0.6246
social media account?	Yes	258 (21.7%)	302 (25.4%)	287 (24.1%)	343 (28.8%)		
Do you live	No	24 (14.4%)	45 (27.0%)	44 (26.4%)	54 (32.3%)	5.945	0.1143
with parent(s)?	Yes	235 (22.7%)	261 (25.2%)	247 (23.8%)	294 (28.4%)		
Previous visits to a	No	250 (22.4%)	287 (25.7%)	276 (24.7%)	303 (27.2%)	24.396	< 0.0001
psychologist/psychiatrist?	Yes	9 (10.2%)	19 (21.6%)	15 (17.1%)	45 (51.1%)		

The results of the statistical analysis reveal significant differences in executive cognition scores based on area of living and sex, both with p-values less than 0.05. Specifically, individuals from rural areas demonstrate a more balanced distribution across the quartiles, with 19.5% in the lowest quartile (Q1) and 27.2% in the highest (Q4). In contrast, urban residents exhibit a tendency towards extremes, with 27.1% in Q1 and 30.0% in Q4, indicating that urban dwellers may experience stronger fluctuations in executive cognition. Additionally, the distribution of scores by sex shows that while males and females have comparable proportions in the lower quartiles, a higher percentage of females (30.6%) fall into the strongest quartile (Q4) compared to males (26.2%). These findings suggest meaningful variations in executive cognition linked to living environment and gender.

## Discussion and conclusion

Social media usage and prolonged engagement with electronic devices have been increasingly recognized as significant factors that detrimentally affect executive functioning in university students, with the literature identifying a range of cognitive and behavioral consequences. Excessive social media use, in particular, has been linked to diminished attention spans, impaired self-regulation, and compromised decision-making abilities, largely due to the fragmented and multitasking-oriented nature of these platforms. Related works have demonstrated that heavy social media consumption can erode cognitive control, making it more difficult for individuals to sustain focus, manage tasks efficiently, and resist impulsive behaviors, all of which are important components of executive functioning [25,26]. Furthermore, the constant influx of notifications and the addictive design of social media platforms exacerbate these issues by fostering a state of continuous partial attention, which undermines the ability to engage in deep, reflective thinking [27,28]. Recent studies have also emphasized the role of digital multitasking, such as switching between academic work and social media, in reducing cognitive performance and increasing mental fatigue, which further impairs executive functions like working memory and cognitive flexibility [29,30]. In addition, the pervasive use of electronic devices, particularly before bedtime, has been shown to disrupt sleep patterns, which in turn negatively affects cognitive processes such as problem-solving, emotional regulation, and inhibitory control [31,32]. These findings are particularly concerning for university students, who often rely on strong executive functioning to manage academic demands, plan long-term projects, and navigate complex social environments. While some studies suggest that moderate and purposeful use of technology can have neutral or even positive effects, the overwhelming evidence points to the need for greater awareness and intervention strategies to mitigate the adverse impacts of excessive digital engagement on cognitive health [33,34]. As the digital landscape continues to evolve, understanding the nuanced relationship between technology use and executive functioning remains an important area of research, particularly in developing evidence-based guidelines to help students balance connectivity with cognitive well-being.

Similarly, poor dietary habits in terms of frequent consumption of junk food, can impair cognitive processes by affecting brain structure and function. Diets high in saturated fats and refined sugars have been linked to reduced hippocampal function, which is important for memory and learning, and are associated with lower cognitive performance overall [35]. This can negatively affect a student’s ability to plan, organize, and problem-solve, which are essential for academic success. Additionally, prolonged use of electronic devices, particularly smartphones and computers, has been shown to contribute to mental fatigue and decreased attention spans. Continuous exposure to screens can interfere with the brain’s ability to process information effectively, further impairing executive functions like working memory, cognitive flexibility, and inhibitory control [31]. Taken together, these lifestyle factors can create a cumulative burden on students’ executive functioning, potentially hindering their academic performance and overall well-being. Therefore, addressing these habits through education and lifestyle interventions is important for enhancing cognitive health and academic outcomes in this population.

Physical activity could play a positive role in enhancing executive functioning in university students, offering numerous cognitive benefits that are supported by a growing body of research. Regular exercise has been shown to improve key aspects of executive function, such as attention, working memory, cognitive flexibility, and inhibitory control. Aerobic exercises, in particular, stimulate the release of neurotrophic factors like brain-derived neurotrophic factor (BDNF), which promotes the growth of new neurons and synaptic plasticity, leading to better cognitive function [36]. Studies also suggest that physical activity enhances prefrontal cortex function, which is the brain region primarily responsible for executive processes, thereby improving students’ ability to regulate emotions, make decisions, and solve complex problems [37]. Furthermore, even short bursts of physical activity, such as a 20-minute walk, have been shown to significantly improve concentration and working memory [38]. Given these findings, incorporating regular physical exercise into the daily routines of university students could serve as an effective intervention to boost executive function, enhance academic performance, and reduce stress.

Research in the literature indicates a significant association between executive function and academic performance, as measured by grade point average (GPA). Students with stronger executive functions often demonstrate better academic outcomes, as these cognitive abilities help them focus, retain information, and organize their learning effectively [39]. A study by McAuley *et al*. [40] found that executive functioning is a significant predictor of academic achievement, with higher executive function scores correlating with better GPA. Additionally, students with deficits in executive function tend to struggle with attention, self-regulation, and planning, which negatively impacts their academic performance [41].

Family income has been shown to influence executive function, particularly in youth and university students. Individuals from lower-income families often face stressors such as financial instability, reduced access to resources, and limited educational support, which can negatively impact the development of executive functions like working memory, attention, and self-regulation. A study by Hackman *et al*. [42] found that socioeconomic status (SES), including family income, is strongly correlated with differences in brain development, particularly in regions associated with executive functioning. Evans and Schamberg [43] further demonstrate that chronic stress in low-income environments contributes to deficits in cognitive performance, including executive function. Conversely, higher-income families tend to provide more cognitively stimulating environments, promoting better executive function development.

Gender, living environment (urban vs. rural), and family dynamics, such as having a strong relationship with extended family, all influence executive function in distinct ways. Research indicates that females often excel in aspects of executive functioning like verbal fluency and emotional regulation, while males may perform better in areas like spatial reasoning and cognitive flexibility [44]. In this study, males slightly outperformed females in executive function scores, though the difference was marginal. This could be attributed to the sensitivity of the assessment tool to tasks that favor males or other factors like cultural influences and stressors. The impact of living environment is also noteworthy–urban settings typically offer greater cognitive stimulation and access to resources that promote cognitive development [45]. While rural environments might offer fewer such opportunities, this study found urban students scored higher in executive function, which could reflect differences in age groups compared to previous research that focused on young children. Additionally, strong family ties, particularly in rural areas, are generally associated with better executive function. These relationships provide emotional support, enhance resilience, and contribute to improved cognitive control and emotional regulation [46].

Previous visits to a psychologist or psychiatrist have been associated with differences in executive function, as these visits often indicate underlying mental health concerns that can impact cognitive processes. Studies have shown that individuals with psychological conditions such as depression, anxiety, or ADHD often experience impairments in executive functioning, including difficulties with attention, planning, and emotional regulation [47,48]. In clinical settings, interventions aimed at treating these mental health conditions can improve executive function over time. In this study, participants who had visited a psychologist or psychiatrist showed higher executive dysfunction scores compared to those who had not, which may reflect the lasting or ongoing cognitive effects of psychological stress or mental health issues that prompted such visits [49].

This study has several limitations that should be acknowledged. First, the inclusion of a broader range of assessment tools could have improved the sensitivity and comprehensiveness of the findings related to specific conditions. Second, incorporating questions about bedtime and sleep duration may provide additional insights into factors that influence executive functioning. Third, the study did not screen for psychiatric and related mood disorders as these significantly influence the responses in DEX and may be misinterpreted as cognitive dysfunction. Fourth, future research could enhance the findings by utilizing a more diverse sample, particularly by comparing students from both public and private universities, and investigating the impact of professional mental health support, such as therapy sessions with psychologists. Fifth, the number of private university students and those who sought mental health services was significantly lower than that of their counterparts. Sixth, the reliance on self-reported surveys may introduce biases, as participants could misrepresent their behaviors, cognitive processes, and emotional states. To mitigate this issue, the survey was conducted online without requiring personal identification, which aimed to minimize response bias. Moreover, given that variations in frontal or executive functioning might affect task completion, email reminders were sent to encourage participant engagement and reduce the potential for non-response bias. Seventh, another limitation pertains to the phrasing of certain questions, especially those related to smartphone usage. Participants’ estimates of usage hours may vary widely, particularly when their usage is both frequent and fragmented (e.g., checking messages multiple times may not translate into clear hourly totals). Eighth, the GPA range could have included students with D or D- grades as a distinct category, which might offer additional insights. Despite these limitations, this study offers valuable insights into the prevalence of executive functioning impairments among university students in Jordan.

## Supporting information

S1 File Output from the statistics software with detailed results. (DOCX)

## References

[1] Landow MV, editor. Stress & mental health of college students. Hauppauge, NY: Nova Science; 2006.

[2] Pedrelli P, Nyer M, Yeung A, Zulauf C, Wilens T. College students: mental health problems and treatment considerations. Acad Psychiatry. 2015;39(5):503–11. doi: 10.1007/s40596-014-0205-9 25142250 PMC4527955

[3] Best JR, Miller PH. A developmental perspective on executive function. Child Dev. 2010;81(6):1641–60. doi: 10.1111/j.1467-8624.2010.01499.x 21077853 PMC3058827

[4] Ahmed SF, Tang S, Waters NE, Davis-Kean P. Executive function and academic achievement: longitudinal relations from early childhood to adolescence. J Educ Psychol. 2019;111(3):446–58. doi: 10.1037/edu0000296

[5] Diamond A. Executive functions. Annu Rev Psychol. 2013;64:135–68. doi: 10.1146/annurev-psych-113011-143750 23020641 PMC4084861

[6] Roselló B, Berenguer C, Baixauli I, Mira Á, Martinez-Raga J, Miranda A. Empirical examination of executive functioning, ADHD associated behaviors, and functional impairments in adults with persistent ADHD, remittent ADHD, and without ADHD. BMC Psychiatry. 2020;20(1):134. doi: 10.1186/s12888-020-02542-y 32204708 PMC7092442

[7] Zelazo PD. Executive function: reflection, iterative reprocessing, complexity, and the developing brain. Dev Rev. 2015;38:55–68. doi: 10.1016/j.dr.2015.07.001

[8] Burgess PW, Alderman N, Evans J, Emslie H, Wilson BA. The ecological validity of tests of executive function. J Int Neuropsychol Soc. 1998;4(6):547–58. doi: 10.1017/s1355617798466037 10050359

[9] Simblett SK, Bateman A. Dimensions of the dysexecutive questionnaire (DEX) examined using Rasch analysis. Neuropsychol Rehabil. 2011;21(1):1–25. doi: 10.1080/09602011.2010.531216 21181602

[10] Bodenburg S, Dopslaff N. The dysexecutive questionnaire advanced: item and test score characteristics, 4-factor solution, and severity classification. J Nerv Ment Dis. 2008;196(1):75–8. doi: 10.1097/NMD.0b013e31815faa2b 18195646

[11] Chaytor N, Schmitter-Edgecombe M, Burr R. Improving the ecological validity of executive functioning assessment. Arch Clin Neuropsychol. 2006;21(3):217–27. doi: 10.1016/j.acn.2005.12.002 16554143

[12] Wilson B, Alderman N, Burgess P, Emslie H, Evans J. Behavioural assessment of the dysexecutive syndrome. Bury, St. Edmunds, UK: Thames Valley Test Company; 1996.

[13] Stuss DT. New approaches to prefrontal lobe testing. In: Miller BL, Cummings JL, editors. The human frontal lobes: functions and disorders. 2nd edition. New York, NY: The Guilford Press; 2007. p. 292–305.

[14] Almomani F, Josman N, Al-Momani MO, Malkawi SH, Nazzal M, Almahdawi KA, et al. Factors related to cognitive function among elementary school children. Scand J Occup Ther. 2014;21(3):191–8. doi: 10.3109/11038128.2013.853098 24215566

[15] Nketia J, Al Sager A, Dajani R, Placido D, Amso D. Executive functions in Jordanian children: what can the hearts and flowers task tell us about development in a non-Western context. J Cognit Dev. 2023;25(2):180–200. doi: 10.1080/15248372.2023.2248698

[16] Wilson B, Evans J, Alderman N, Burgess P, Emslie H. Methodologies and models in the study of executive function. In: Rabbitt P, editor. Methodology of frontal and executive function. East Sussex, England: Psychology Press; 1997. p. 1–38.

[17] Simblett SK, Ring H, Bateman A. The Dysexecutive Questionnaire Revised (DEX-R): an extended measure of everyday dysexecutive problems after acquired brain injury. Neuropsychol Rehabil. 2017;27(8):1124–41. doi: 10.1080/09602011.2015.1121880 26784858

[18] Cervantes-Cardona GA, Nápoles-Echauri A, Alonso-Estrella N, Hernández-Mora FJ, Cervantes-Pérez E, Cervantes-Guevara G, et al. Prevalence of dysexecutive symptoms in high school students during the COVID-19 pandemic. Int J Environ Res Public Health. 2022;19(23):15641. doi: 10.3390/ijerph192315641 36497715 PMC9740397

[19] Chan RCK. Dysexecutive symptoms among a non-clinical sample: a study with the use of the Dysexecutive Questionnaire. Br J Psychol. 2001;92(3):551–65. doi: 10.1348/000712601162338 11802889

[20] Bennett PC, Ong B, Ponsford J. Measuring executive dysfunction in an acute rehabilitation setting: using the dysexecutive questionnaire (DEX). J Int Neuropsychol Soc. 2005;11(4):376–85. doi: 10.1017/s1355617705050423 16209417

[21] Pedrero Pérez EJ, Ruiz Sánchez De León JM, Rojo Mota G, Llanero Luque M, Olivar Arroyo Á, Bouso Saiz JC, et al. Versión española del Cuestionario Disejecutivo (DEX-Sp): propiedades psicométricas en adictos y población no clínica. Adicciones. 2009;21(2):155–66. doi: 10.20882/adicciones.243 19578733

[22] Shaw S, Oei TPS, Sawang S. Psychometric validation of the dysexecutive questionnaire (DEX). Psychol Assess. 2015;27(1):138–47. doi: 10.1037/a0038195 25602692

[23] Sperber AD, Devellis RF, Boehlecke B. Cross-cultural translation. J Cross-Cultural Psychol. 1994;25(4):501–24. doi: 10.1177/0022022194254006

[24] Statistical software. Available from: https://www.jmp.com/en_us/home.html

[25] Martín-Perpiñá MM, Viñas Poch F, Malo Cerrato S. Media multitasking impact in homework, executive function and academic performance in Spanish adolescents. Psicothema. 2019;31(1):81–7. doi: 10.7334/psicothema2018.178 30664415

[26] Luo J, Li H, Yeung P, Chang C. The association between media multitasking and executive function in Chinese adolescents: evidence from self-reported, behavioral and fNIRS data. Cyberpsychology. 2021;15(2). doi: 10.5817/cp2021-2-8

[27] Cain MS, Leonard JA, Gabrieli JDE, Finn AS. Media multitasking in adolescence. Psychon Bull Rev. 2016;23(6):1932–41. doi: 10.3758/s13423-016-1036-3 27188785

[28] Montag C, Walla P. Carpe diem instead of losing your social mind: beyond digital addiction and why we all suffer from digital overuse. Cogent Psychol. 2016;3(1):1157281. doi: 10.1080/23311908.2016.1157281

[29] Mark G, Gonzalez VM, Harris J. No task left behind? In: Proceedings of the SIGCHI conference on human factors in computing systems. ACM Press; 2005. p. 321–30. doi: 10.1145/1054972.1055017

[30] Rosen LD, Mark Carrier L, Cheever NA. Facebook and texting made me do it: media-induced task-switching while studying. Comput Hum Behav. 2013;29(3):948–58. doi: 10.1016/j.chb.2012.12.001

[31] Twenge JM, Joiner TE, Rogers ML, Martin GN. Increases in depressive symptoms, suicide-related outcomes, and suicide rates among U.S. adolescents after 2010 and links to increased new media screen time. Clin Psychol Sci. 2017;6(1):3–17. doi: 10.1177/2167702617723376

[32] Alonzo R, Hussain J, Stranges S, Anderson KK. Interplay between social media use, sleep quality, and mental health in youth: a systematic review. Sleep Med Rev. 2021;56:101414. doi: 10.1016/j.smrv.2020.101414 33385767

[33] Przybylski AK, Weinstein N. Digital screen time limits and young children’s psychological well-being: evidence from a population-based study. Child Dev. 2019;90(1):e56–e65. doi: 10.1111/cdev.13007 29235663

[34] van der Schuur WA, Baumgartner SE, Sumter SR, Valkenburg PM. The consequences of media multitasking for youth: a review. Comput Hum Behav. 2015;53:204–15. doi: 10.1016/j.chb.2015.06.035

[35] Gómez-Pinilla F. Brain foods: the effects of nutrients on brain function. Nat Rev Neurosci. 2008;9(7):568–78. doi: 10.1038/nrn2421 18568016 PMC2805706

[36] Erickson KI, Voss MW, Prakash RS, Basak C, Szabo A, Chaddock L, et al. Exercise training increases size of hippocampus and improves memory. Proc Natl Acad Sci U S A. 2011;108(7):3017–22. doi: 10.1073/pnas.1015950108 21282661 PMC3041121

[37] Best JR. Effects of physical activity on children’s executive function: contributions of experimental research on aerobic exercise. Dev Rev. 2010;30(4):331–551. doi: 10.1016/j.dr.2010.08.001 21818169 PMC3147174

[38] Hillman CH, Pontifex MB, Raine LB, Castelli DM, Hall EE, Kramer AF. The effect of acute treadmill walking on cognitive control and academic achievement in preadolescent children. Neuroscience. 2009;159(3):1044–54. doi: 10.1016/j.neuroscience.2009.01.057 19356688 PMC2667807

[39] Best JR, Miller PH, Naglieri JA. Relations between executive function and academic achievement from ages 5 to 17 in a large, representative national sample. Learn Individ Differ. 2011;21(4):327–36. doi: 10.1016/j.lindif.2011.01.007 21845021 PMC3155246

[40] Mcauley T, Chen S, Goos L, Schachar R, Crosbie J. Is the behavior rating inventory of executive function more strongly associated with measures of impairment or executive function? J Int Neuropsychol Soc. 2010;16(3):495–505. doi: 10.1017/s1355617710000093 20188014

[41] Duckworth AL, Seligman MEP. Self-discipline outdoes IQ in predicting academic performance of adolescents. Psychol Sci. 2005;16(12):939–44. doi: 10.1111/j.1467-9280.2005.01641.x 16313657

[42] Hackman DA, Farah MJ, Meaney MJ. Socioeconomic status and the brain: mechanistic insights from human and animal research. Nat Rev Neurosci. 2010;11(9):651–9. doi: 10.1038/nrn2897 20725096 PMC2950073

[43] Evans GW, Schamberg MA. Childhood poverty, chronic stress, and adult working memory. Proc Natl Acad Sci U S A. 2009;106(16):6545–9. doi: 10.1073/pnas.0811910106 19332779 PMC2662958

[44] Gur RC, Turetsky BI, Matsui M, Yan M, Bilker W, Hughett P, et al. Sex differences in brain gray and white matter in healthy young adults: correlations with cognitive performance. J Neurosci. 1999;19(10):4065–72. doi: 10.1523/JNEUROSCI.19-10-04065.1999 10234034 PMC6782697

[45] Grantham-McGregor S, Cheung YB, Cueto S, Glewwe P, Richter L, Strupp B, et al. Developmental potential in the first 5 years for children in developing countries. Lancet. 2007;369(9555):60–70. doi: 10.1016/S0140-6736(07)60032-4 17208643 PMC2270351

[46] Taylor RD, Oberle E, Durlak JA, Weissberg RP. Promoting positive youth development through school-based social and emotional learning interventions: a meta-analysis of follow-up effects. Child Dev. 2017;88(4):1156–71. doi: 10.1111/cdev.12864 28685826

[47] Castaneda AE, Tuulio-Henriksson A, Marttunen M, Suvisaari J, Lönnqvist J. A review on cognitive impairments in depressive and anxiety disorders with a focus on young adults. J Affect Disord. 2008;106(1–2):1–27. doi: 10.1016/j.jad.2007.06.006 17707915

[48] Christian C, Martel MM, Levinson CA. Emotion regulation difficulties, but not negative urgency, are associated with attention-deficit/hyperactivity disorder and eating disorder symptoms in undergraduate students. Eat Behav. 2020;36:101344. doi: 10.1016/j.eatbeh.2019.101344 31743854

[49] Gillespie ML, Rao U. Relationships between depression and executive functioning in adolescents: the moderating role of unpredictable home environment. J Child Fam Stud. 2022;31(9):2518–34. doi: 10.1007/s10826-022-02296-z 36504694 PMC9733726

