# Supplementary material for: Prevalence and contributing factors of executive cognitive dysfunction symptoms in university students
Source: PLoS One. 2025 Jun 9;20(6):e0323783. doi: 10.1371/journal.pone.0323783 (PMC12148170; doi:10.1371/journal.pone.0323783)
Supplement: S1 File — (DOCX) [file pone.0323783.s001.docx]

**Fit Group**

**Oneway Analysis of Executive Cognition By هل سبق و ان راجعت مختص بالصحة النفسيه؟**

**Oneway Anova**

**Summary of Fit**

| Rsquare | 0.01327 |
| --- | --- |
| Adj Rsquare | 0.012449 |
| Root Mean Square Error | 7.16654 |
| Mean of Response | 25.31561 |
| Observations (or Sum Wgts) | 1204 |

**Pooled t Test**

2-1

Assuming equal variances

| Difference | 3.19029 | t Ratio | 4.020507 |
| --- | --- | --- | --- |
| Std Err Dif | 0.79350 | DF | 1202 |
| Upper CL Dif | 4.74710 | Prob > \|t\| | <.0001* |
| Lower CL Dif | 1.63348 | Prob > t | <.0001* |
| Confidence | 0.95 | Prob < t | 1.0000 |

**Analysis of Variance**

| **Source** | **DF** | **Sum of Squares** | **Mean Square** | **F Ratio** | **Prob > F** |
| --- | --- | --- | --- | --- | --- |
| هل سبق و ان راجعت مختص بالصحة النفسيه؟ | 1 | 830.196 | 830.196 | 16.1645 | <.0001* |
| Error | 1202 | 61733.870 | 51.359 |  |  |
| C. Total | 1203 | 62564.066 |  |  |  |

**Means for Oneway Anova**

| **Level** | **Number** | **Mean** | **Std Error** | **Lower 95%** | **Upper 95%** |
| --- | --- | --- | --- | --- | --- |
| 1 | 1116 | 25.0824 | 0.21452 | 24.662 | 25.503 |
| 2 | 88 | 28.2727 | 0.76396 | 26.774 | 29.772 |

Std Error uses a pooled estimate of error variance

**Means and Std Deviations**

| **Level** | **Number** | **Mean** | **Std Dev** | **Std Err Mean** | **Lower 95%** | **Upper 95%** | **Std Dev Lower 95%** | **Std Dev Upper 95%** | **** |
| --- | --- | --- | --- | --- | --- | --- | --- | --- | --- |
| 1 | 1116 | 25.082437 | 7.1870846 | 0.2151397 | 24.660313 | 25.504562 | 6.9007928 | 7.4983423 |  |
| 2 | 88 | 28.272727 | 6.8978212 | 0.7353102 | 26.811219 | 29.734236 | 6.0076304 | 8.1001587 |  |

| Missing Rows | 1 |
| --- | --- |
| Excluded Rows | 5 |

**Oneway Analysis of Executive Cognition By مجموع دخل ا1سرة الشهري**

**Oneway Anova**

**Summary of Fit**

| Rsquare | 0.008436 |
| --- | --- |
| Adj Rsquare | 0.005957 |
| Root Mean Square Error | 7.190055 |
| Mean of Response | 25.31561 |
| Observations (or Sum Wgts) | 1204 |

**Analysis of Variance**

| **Source** | **DF** | **Sum of Squares** | **Mean Square** | **F Ratio** | **Prob > F** |
| --- | --- | --- | --- | --- | --- |
| مجموع دخل ا1سرة الشهري | 3 | 527.796 | 175.932 | 3.4031 | 0.0172* |
| Error | 1200 | 62036.270 | 51.697 |  |  |
| C. Total | 1203 | 62564.066 |  |  |  |

**Means for Oneway Anova**

| **Level** | **Number** | **Mean** | **Std Error** | **Lower 95%** | **Upper 95%** |
| --- | --- | --- | --- | --- | --- |
| 1 | 731 | 25.2599 | 0.26593 | 24.738 | 25.782 |
| 2 | 333 | 24.7568 | 0.39401 | 23.984 | 25.530 |
| 3 | 70 | 26.3143 | 0.85938 | 24.628 | 28.000 |
| 4 | 70 | 27.5571 | 0.85938 | 25.871 | 29.243 |

Std Error uses a pooled estimate of error variance

**Means and Std Deviations**

| **Level** | **Number** | **Mean** | **Std Dev** | **Std Err Mean** | **Lower 95%** | **Upper 95%** | **Std Dev Lower 95%** | **Std Dev Upper 95%** | **** |
| --- | --- | --- | --- | --- | --- | --- | --- | --- | --- |
| 1 | 731 | 25.259918 | 7.4236401 | 0.2745732 | 24.720871 | 25.798965 | 7.061623 | 7.8250739 |  |
| 2 | 333 | 24.756757 | 6.7389573 | 0.3692926 | 24.030308 | 25.483205 | 6.2630536 | 7.2937357 |  |
| 3 | 70 | 26.314286 | 6.4505766 | 0.7709914 | 24.7762 | 27.852371 | 5.5308384 | 7.7400865 |  |
| 4 | 70 | 27.557143 | 7.4767964 | 0.8936481 | 25.774364 | 29.339922 | 6.4107374 | 8.971454 |  |

**Means Comparisons**

**Comparisons for all pairs using Tukey-Kramer HSD**

**Confidence Quantile**

| **q*** | **Alpha** |
| --- | --- |
| 2.57270 | 0.05 |

**HSD Threshold Matrix**

Abs(Dif)-HSD

|  | **4** | **3** | **1** | **2** |
| --- | --- | --- | --- | --- |
| 4 | -3.1267 | -1.8839 | -0.0171 | 0.3682 |
| 3 | -1.8839 | -3.1267 | -1.2600 | -0.8747 |
| 1 | -0.0171 | -1.2600 | -0.9676 | -0.7198 |
| 2 | 0.3682 | -0.8747 | -0.7198 | -1.4336 |

Positive values show pairs of means that are significantly different.

**Connecting Letters Report**

| **Level** |  |  | **Mean** |
| --- | --- | --- | --- |
| 4 | A |  | 27.557143 |
| 3 | A | B | 26.314286 |
| 1 | A | B | 25.259918 |
| 2 |  | B | 24.756757 |

Levels not connected by same letter are significantly different.

**Ordered Differences Report**

| **Level** | **- Level** | **Difference** | **Std Err Dif** | **Lower CL** | **Upper CL** | **p-Value** | **** |
| --- | --- | --- | --- | --- | --- | --- | --- |
| 4 | 2 | 2.800386 | 0.945396 | 0.36817 | 5.232605 | 0.0164* |  |
| 4 | 1 | 2.297225 | 0.899582 | -0.01713 | 4.611579 | 0.0526 |  |
| 3 | 2 | 1.557529 | 0.945396 | -0.87469 | 3.989748 | 0.3524 |  |
| 4 | 3 | 1.242857 | 1.215341 | -1.88385 | 4.369565 | 0.7362 |  |
| 3 | 1 | 1.054368 | 0.899582 | -1.25999 | 3.368722 | 0.6446 |  |
| 1 | 2 | 0.503161 | 0.475360 | -0.71980 | 1.726119 | 0.7149 |  |

| Missing Rows | 1 |
| --- | --- |
| Excluded Rows | 5 |

**Oneway Analysis of Executive Cognition By مكان السكن**

**Oneway Anova**

**Summary of Fit**

| Rsquare | 0.004683 |
| --- | --- |
| Adj Rsquare | 0.003855 |
| Root Mean Square Error | 7.197655 |
| Mean of Response | 25.31561 |
| Observations (or Sum Wgts) | 1204 |

**Pooled t Test**

2-1

Assuming equal variances

| Difference | 1.01235 | t Ratio | 2.378026 |
| --- | --- | --- | --- |
| Std Err Dif | 0.42571 | DF | 1202 |
| Upper CL Dif | 1.84756 | Prob > \|t\| | 0.0176* |
| Lower CL Dif | 0.17713 | Prob > t | 0.0088* |
| Confidence | 0.95 | Prob < t | 0.9912 |

**Analysis of Variance**

| **Source** | **DF** | **Sum of Squares** | **Mean Square** | **F Ratio** | **Prob > F** |
| --- | --- | --- | --- | --- | --- |
| مكان السكن | 1 | 292.965 | 292.965 | 5.6550 | 0.0176* |
| Error | 1202 | 62271.102 | 51.806 |  |  |
| C. Total | 1203 | 62564.066 |  |  |  |

**Means for Oneway Anova**

| **Level** | **Number** | **Mean** | **Std Error** | **Lower 95%** | **Upper 95%** |
| --- | --- | --- | --- | --- | --- |
| 1 | 467 | 24.6959 | 0.33307 | 24.042 | 25.349 |
| 2 | 737 | 25.7083 | 0.26513 | 25.188 | 26.228 |

Std Error uses a pooled estimate of error variance

**Means and Std Deviations**

| **Level** | **Number** | **Mean** | **Std Dev** | **Std Err Mean** | **Lower 95%** | **Upper 95%** | **Std Dev Lower 95%** | **Std Dev Upper 95%** | **** |
| --- | --- | --- | --- | --- | --- | --- | --- | --- | --- |
| 1 | 467 | 24.695931 | 7.3969055 | 0.342288 | 24.023312 | 25.368551 | 6.9509935 | 7.9044157 |  |
| 2 | 737 | 25.708277 | 7.0685963 | 0.2603751 | 25.19711 | 26.219443 | 6.7252316 | 7.449186 |  |

| Missing Rows | 1 |
| --- | --- |
| Excluded Rows | 5 |

**Oneway Analysis of Executive Cognition By هل تشعر أن ع1قتك بالعائلة الممتدة (أبناء ا1خوال أو ا1عمام) قوية ؟**

**Oneway Anova**

**Summary of Fit**

| Rsquare | 0.004649 |
| --- | --- |
| Adj Rsquare | 0.003821 |
| Root Mean Square Error | 7.197776 |
| Mean of Response | 25.31561 |
| Observations (or Sum Wgts) | 1204 |

**Pooled t Test**

2-1

Assuming equal variances

| Difference | -1.0449 | t Ratio | -2.36948 |
| --- | --- | --- | --- |
| Std Err Dif | 0.4410 | DF | 1202 |
| Upper CL Dif | -0.1797 | Prob > \|t\| | 0.0180* |
| Lower CL Dif | -1.9100 | Prob > t | 0.9910 |
| Confidence | 0.95 | Prob < t | 0.0090* |

**Analysis of Variance**

| **Source** | **DF** | **Sum of Squares** | **Mean Square** | **F Ratio** | **Prob > F** |
| --- | --- | --- | --- | --- | --- |
| هل تشعر أن ع1قتك بالعائلة الممتدة (أبناء ا1خوال أو ا1عمام) قوية ؟ | 1 | 290.872 | 290.872 | 5.6144 | 0.0180* |
| Error | 1202 | 62273.195 | 51.808 |  |  |
| C. Total | 1203 | 62564.066 |  |  |  |

**Means for Oneway Anova**

| **Level** | **Number** | **Mean** | **Std Error** | **Lower 95%** | **Upper 95%** |
| --- | --- | --- | --- | --- | --- |
| 1 | 398 | 26.0151 | 0.36079 | 25.307 | 26.723 |
| 2 | 806 | 24.9702 | 0.25353 | 24.473 | 25.468 |

Std Error uses a pooled estimate of error variance

**Means and Std Deviations**

| **Level** | **Number** | **Mean** | **Std Dev** | **Std Err Mean** | **Lower 95%** | **Upper 95%** | **Std Dev Lower 95%** | **Std Dev Upper 95%** | **** |
| --- | --- | --- | --- | --- | --- | --- | --- | --- | --- |
| 1 | 398 | 26.015075 | 6.9103488 | 0.3463845 | 25.334098 | 26.696053 | 6.4613141 | 7.4269704 |  |
| 2 | 806 | 24.970223 | 7.3353806 | 0.2583778 | 24.46305 | 25.477397 | 6.9939319 | 7.7121416 |  |

| Missing Rows | 1 |
| --- | --- |
| Excluded Rows | 5 |

**Oneway Analysis of Executive Cognition By الجنس**

**Oneway Anova**

**Summary of Fit**

| Rsquare | 0.003962 |
| --- | --- |
| Adj Rsquare | 0.003133 |
| Root Mean Square Error | 7.200262 |
| Mean of Response | 25.31561 |
| Observations (or Sum Wgts) | 1204 |

**Pooled t Test**

2-1

Assuming equal variances

| Difference | 0.93152 | t Ratio | 2.186504 |
| --- | --- | --- | --- |
| Std Err Dif | 0.42603 | DF | 1202 |
| Upper CL Dif | 1.76736 | Prob > \|t\| | 0.0290* |
| Lower CL Dif | 0.09567 | Prob > t | 0.0145* |
| Confidence | 0.95 | Prob < t | 0.9855 |

**Analysis of Variance**

| **Source** | **DF** | **Sum of Squares** | **Mean Square** | **F Ratio** | **Prob > F** |
| --- | --- | --- | --- | --- | --- |
| الجنس | 1 | 247.855 | 247.855 | 4.7808 | 0.0290* |
| Error | 1202 | 62316.212 | 51.844 |  |  |
| C. Total | 1203 | 62564.066 |  |  |  |

**Means for Oneway Anova**

| **Level** | **Number** | **Mean** | **Std Error** | **Lower 95%** | **Upper 95%** |
| --- | --- | --- | --- | --- | --- |
| 1 | 466 | 24.7446 | 0.33355 | 24.090 | 25.399 |
| 2 | 738 | 25.6762 | 0.26505 | 25.156 | 26.196 |

Std Error uses a pooled estimate of error variance

**Means and Std Deviations**

| **Level** | **Number** | **Mean** | **Std Dev** | **Std Err Mean** | **Lower 95%** | **Upper 95%** | **Std Dev Lower 95%** | **Std Dev Upper 95%** | **** |
| --- | --- | --- | --- | --- | --- | --- | --- | --- | --- |
| 1 | 466 | 24.744635 | 7.6260182 | 0.3532685 | 24.050435 | 25.438836 | 7.1658308 | 8.1498489 |  |
| 2 | 738 | 25.676152 | 6.9181687 | 0.2546613 | 25.176204 | 26.1761 | 6.582328 | 7.2903927 |  |

| Missing Rows | 1 |
| --- | --- |
| Excluded Rows | 5 |

**Oneway Analysis of Executive Cognition By هل ا1ب و ا1م مطلقين من بعض؟**

**Oneway Anova**

**Summary of Fit**

| Rsquare | 0.003596 |
| --- | --- |
| Adj Rsquare | 0.002767 |
| Root Mean Square Error | 7.201584 |
| Mean of Response | 25.31561 |
| Observations (or Sum Wgts) | 1204 |

**Pooled t Test**

2-1

Assuming equal variances

| Difference | 2.53805 | t Ratio | 2.082725 |
| --- | --- | --- | --- |
| Std Err Dif | 1.21862 | DF | 1202 |
| Upper CL Dif | 4.92891 | Prob > \|t\| | 0.0375* |
| Lower CL Dif | 0.14719 | Prob > t | 0.0187* |
| Confidence | 0.95 | Prob < t | 0.9813 |

**Analysis of Variance**

| **Source** | **DF** | **Sum of Squares** | **Mean Square** | **F Ratio** | **Prob > F** |
| --- | --- | --- | --- | --- | --- |
| هل ا1ب و ا1م مطلقين من بعض؟ | 1 | 224.968 | 224.968 | 4.3377 | 0.0375* |
| Error | 1202 | 62339.099 | 51.863 |  |  |
| C. Total | 1203 | 62564.066 |  |  |  |

**Means for Oneway Anova**

| **Level** | **Number** | **Mean** | **Std Error** | **Lower 95%** | **Upper 95%** |
| --- | --- | --- | --- | --- | --- |
| 1 | 1168 | 25.2397 | 0.2107 | 24.826 | 25.653 |
| 2 | 36 | 27.7778 | 1.2003 | 25.423 | 30.133 |

Std Error uses a pooled estimate of error variance

**Means and Std Deviations**

| **Level** | **Number** | **Mean** | **Std Dev** | **Std Err Mean** | **Lower 95%** | **Upper 95%** | **Std Dev Lower 95%** | **Std Dev Upper 95%** | **** |
| --- | --- | --- | --- | --- | --- | --- | --- | --- | --- |
| 1 | 1168 | 25.239726 | 7.2361842 | 0.2117328 | 24.824306 | 25.655146 | 6.9541752 | 7.5422083 |  |
| 2 | 36 | 27.777778 | 5.9334938 | 0.9889156 | 25.770172 | 29.785383 | 4.8125476 | 7.7398751 |  |

| Missing Rows | 1 |
| --- | --- |
| Excluded Rows | 5 |

**Oneway Analysis of Executive Cognition By هل يسكن الطالب مع اهله ام في 1؟**

**Oneway Anova**

**Summary of Fit**

| Rsquare | 0.003073 |
| --- | --- |
| Adj Rsquare | 0.002244 |
| Root Mean Square Error | 7.203473 |
| Mean of Response | 25.31561 |
| Observations (or Sum Wgts) | 1204 |

**Pooled t Test**

2-1

Assuming equal variances

| Difference | -1.1561 | t Ratio | -1.92485 |
| --- | --- | --- | --- |
| Std Err Dif | 0.6006 | DF | 1202 |
| Upper CL Dif | 0.0223 | Prob > \|t\| | 0.0545 |
| Lower CL Dif | -2.3345 | Prob > t | 0.9728 |
| Confidence | 0.95 | Prob < t | 0.0272* |

**Analysis of Variance**

| **Source** | **DF** | **Sum of Squares** | **Mean Square** | **F Ratio** | **Prob > F** |
| --- | --- | --- | --- | --- | --- |
| هل يسكن الطالب مع اهله ام في 1؟ | 1 | 192.254 | 192.254 | 3.7050 | 0.0545 |
| Error | 1202 | 62371.812 | 51.890 |  |  |
| C. Total | 1203 | 62564.066 |  |  |  |

**Means for Oneway Anova**

| **Level** | **Number** | **Mean** | **Std Error** | **Lower 95%** | **Upper 95%** |
| --- | --- | --- | --- | --- | --- |
| 1 | 167 | 26.3114 | 0.55742 | 25.218 | 27.405 |
| 2 | 1037 | 25.1553 | 0.22369 | 24.716 | 25.594 |

Std Error uses a pooled estimate of error variance

**Means and Std Deviations**

| **Level** | **Number** | **Mean** | **Std Dev** | **Std Err Mean** | **Lower 95%** | **Upper 95%** | **Std Dev Lower 95%** | **Std Dev Upper 95%** | **** |
| --- | --- | --- | --- | --- | --- | --- | --- | --- | --- |
| 1 | 167 | 26.311377 | 6.9783695 | 0.5400024 | 25.245219 | 27.377535 | 6.3016468 | 7.8192049 |  |
| 2 | 1037 | 25.155256 | 7.2388914 | 0.2247929 | 24.714154 | 25.596357 | 6.9401951 | 7.5646539 |  |

| Missing Rows | 1 |
| --- | --- |
| Excluded Rows | 5 |

**Oneway Analysis of Executive Cognition By جامعة**

**Oneway Anova**

**Summary of Fit**

| Rsquare | 0.002459 |
| --- | --- |
| Adj Rsquare | 0.001629 |
| Root Mean Square Error | 7.205692 |
| Mean of Response | 25.31561 |
| Observations (or Sum Wgts) | 1204 |

**Pooled t Test**

2-1

Assuming equal variances

| Difference | -1.3258 | t Ratio | -1.7212 |
| --- | --- | --- | --- |
| Std Err Dif | 0.7703 | DF | 1202 |
| Upper CL Dif | 0.1854 | Prob > \|t\| | 0.0855 |
| Lower CL Dif | -2.8371 | Prob > t | 0.9573 |
| Confidence | 0.95 | Prob < t | 0.0427* |

**Analysis of Variance**

| **Source** | **DF** | **Sum of Squares** | **Mean Square** | **F Ratio** | **Prob > F** |
| --- | --- | --- | --- | --- | --- |
| جامعة | 1 | 153.820 | 153.820 | 2.9625 | 0.0855 |
| Error | 1202 | 62410.247 | 51.922 |  |  |
| C. Total | 1203 | 62564.066 |  |  |  |

**Means for Oneway Anova**

| **Level** | **Number** | **Mean** | **Std Error** | **Lower 95%** | **Upper 95%** |
| --- | --- | --- | --- | --- | --- |
| 1 | 95 | 26.5368 | 0.73929 | 25.086 | 27.987 |
| 2 | 1109 | 25.2110 | 0.21638 | 24.786 | 25.636 |

Std Error uses a pooled estimate of error variance

**Means and Std Deviations**

| **Level** | **Number** | **Mean** | **Std Dev** | **Std Err Mean** | **Lower 95%** | **Upper 95%** | **Std Dev Lower 95%** | **Std Dev Upper 95%** | **** |
| --- | --- | --- | --- | --- | --- | --- | --- | --- | --- |
| 1 | 95 | 26.536842 | 7.6266756 | 0.7824804 | 24.983209 | 28.090475 | 6.6749834 | 8.8973734 |  |
| 2 | 1109 | 25.211001 | 7.1688396 | 0.2152698 | 24.788618 | 25.633383 | 6.8824105 | 7.4803289 |  |

| Missing Rows | 1 |
| --- | --- |
| Excluded Rows | 5 |

**Bivariate Fit of Executive Cognition By أي من وسائل التواصل التوصل ا1جتماعي تستخدم غالباً (ممكن اكثر من اختيار) ؟ 2**

**Linear Fit**

Executive Cognition = 24.583763 + 0.3413983*أي من وسائل التواصل التوصل ا1جتماعي تستخدم غالباً (ممكن اكثر من اختيار) ؟ 2

**Summary of Fit**

| RSquare | 0.003238 |
| --- | --- |
| RSquare Adj | 0.002409 |
| Root Mean Square Error | 7.202877 |
| Mean of Response | 25.31561 |
| Observations (or Sum Wgts) | 1204 |

**Analysis of Variance**

| **Source** | **DF** | **Sum of Squares** | **Mean Square** | **F Ratio** |
| --- | --- | --- | --- | --- |
| Model | 1 | 202.585 | 202.585 | 3.9048 |
| Error | 1202 | 62361.481 | 51.881 | **Prob > F** |
| C. Total | 1203 | 62564.066 |  | 0.0484* |

**Parameter Estimates**

| **Term** | **Estimate** | **Std Error** | **t Ratio** | **Prob>\|t\|** |
| --- | --- | --- | --- | --- |
| Intercept | 24.583763 | 0.424568 | 57.90 | <.0001* |
| أي من وسائل التواصل التوصل ا1جتماعي تستخدم غالباً (ممكن اكثر من اختيار) ؟ 2 | 0.3413983 | 0.172768 | 1.98 | 0.0484* |

**Bivariate Fit of Executive Cognition By كم 1تستخدم وسائل التواصل ا1جتماعي؟ 2**

**Linear Fit**

Executive Cognition = 23.060538 + 0.4885937*كم 1تستخدم وسائل التواصل ا1جتماعي؟ 2

**Summary of Fit**

| RSquare | 0.02198 |
| --- | --- |
| RSquare Adj | 0.021114 |
| Root Mean Square Error | 7.175797 |
| Mean of Response | 25.1864 |
| Observations (or Sum Wgts) | 1132 |

**Analysis of Variance**

| **Source** | **DF** | **Sum of Squares** | **Mean Square** | **F Ratio** |
| --- | --- | --- | --- | --- |
| Model | 1 | 1307.645 | 1307.65 | 25.3951 |
| Error | 1130 | 58186.025 | 51.49 | **Prob > F** |
| C. Total | 1131 | 59493.670 |  | <.0001* |

**Parameter Estimates**

| **Term** | **Estimate** | **Std Error** | **t Ratio** | **Prob>\|t\|** |
| --- | --- | --- | --- | --- |
| Intercept | 23.060538 | 0.472701 | 48.78 | <.0001* |
| كم 1تستخدم وسائل التواصل ا1جتماعي؟ 2 | 0.4885937 | 0.096956 | 5.04 | <.0001* |

**Bivariate Fit of Executive Cognition By كم تمارس الرياضه اسبوعيا؟ 3**

**Linear Fit**

Executive Cognition = 25.880954 - 0.1641576*كم تمارس الرياضه اسبوعيا؟ 3

**Summary of Fit**

| RSquare | 0.003966 |
| --- | --- |
| RSquare Adj | 0.003098 |
| Root Mean Square Error | 7.237018 |
| Mean of Response | 25.3887 |
| Observations (or Sum Wgts) | 1150 |

**Analysis of Variance**

| **Source** | **DF** | **Sum of Squares** | **Mean Square** | **F Ratio** |
| --- | --- | --- | --- | --- |
| Model | 1 | 239.410 | 239.410 | 4.5711 |
| Error | 1148 | 60125.843 | 52.374 | **Prob > F** |
| C. Total | 1149 | 60365.253 |  | 0.0327* |

**Parameter Estimates**

| **Term** | **Estimate** | **Std Error** | **t Ratio** | **Prob>\|t\|** |
| --- | --- | --- | --- | --- |
| Intercept | 25.880954 | 0.313933 | 82.44 | <.0001* |
| كم تمارس الرياضه اسبوعيا؟ 3 | -0.164158 | 0.07678 | -2.14 | 0.0327* |

**Bivariate Fit of Executive Cognition By كم مرة تأكل الوجبات السريعة أسبوعيا؟**

**Linear Fit**

Executive Cognition = 24.1296 + 0.5639659*كم مرة تأكل الوجبات السريعة أسبوعيا؟

**Summary of Fit**

| RSquare | 0.012736 |
| --- | --- |
| RSquare Adj | 0.011915 |
| Root Mean Square Error | 7.168478 |
| Mean of Response | 25.31561 |
| Observations (or Sum Wgts) | 1204 |

**Analysis of Variance**

| **Source** | **DF** | **Sum of Squares** | **Mean Square** | **F Ratio** |
| --- | --- | --- | --- | --- |
| Model | 1 | 796.807 | 796.807 | 15.5060 |
| Error | 1202 | 61767.259 | 51.387 | **Prob > F** |
| C. Total | 1203 | 62564.066 |  | <.0001* |

**Parameter Estimates**

| **Term** | **Estimate** | **Std Error** | **t Ratio** | **Prob>\|t\|** |
| --- | --- | --- | --- | --- |
| Intercept | 24.1296 | 0.365234 | 66.07 | <.0001* |
| كم مرة تأكل الوجبات السريعة أسبوعيا؟ | 0.5639659 | 0.14322 | 3.94 | <.0001* |

**Bivariate Fit of Executive Cognition By كم تستخدم الهاتف و ا1جهزه ا1لكترونيه يوميا؟**

**Linear Fit**

Executive Cognition = 22.370657 + 0.4683474*كم تستخدم الهاتف و ا1جهزه ا1لكترونيه يوميا؟

**Summary of Fit**

| RSquare | 0.020578 |
| --- | --- |
| RSquare Adj | 0.01964 |
| Root Mean Square Error | 7.099635 |
| Mean of Response | 25.08604 |
| Observations (or Sum Wgts) | 1046 |

**Analysis of Variance**

| **Source** | **DF** | **Sum of Squares** | **Mean Square** | **F Ratio** |
| --- | --- | --- | --- | --- |
| Model | 1 | 1105.627 | 1105.63 | 21.9349 |
| Error | 1044 | 52622.630 | 50.40 | **Prob > F** |
| C. Total | 1045 | 53728.256 |  | <.0001* |

**Parameter Estimates**

| **Term** | **Estimate** | **Std Error** | **t Ratio** | **Prob>\|t\|** |
| --- | --- | --- | --- | --- |
| Intercept | 22.370657 | 0.619946 | 36.08 | <.0001* |
| كم تستخدم الهاتف و ا1جهزه ا1لكترونيه يوميا؟ | 0.4683474 | 0.1 | 4.68 | <.0001* |
